# An EdU-based flow cytometry assay to evaluate chicken T lymphocyte proliferation

**DOI:** 10.1186/s12917-020-02433-0

**Published:** 2020-07-06

**Authors:** Karla Lucía F. Alvarez, Astrid Poma-Acevedo, Manolo Fernández-Sánchez, Manolo Fernández-Díaz

**Affiliations:** Research and Development Laboratories, FARVET, Carretera Panamericana Sur N°766 Km 198.5, Ica, Peru

**Keywords:** EdU proliferation assay, Chicken T lymphocytes, Poultry industry

## Abstract

**Background:**

In the poultry industry, quantitative analysis of chicken T cell proliferation is important in many biological applications such as drug screening, vaccine production, and cytotoxicity assessment. Several assays have been established to evaluate this immunological response in chicken cells. However, these assays have some disadvantages including use of radioactive labels ([3H]-Thymidine assay), necessity of DNA denaturation or digestion (BrdU incorporation assay), lack of sensitivity and underestimation of anti-proliferative effects (MTT assay), and modulation of activation molecules and cell viability reduction (CFSE assay). Overcoming these limitations, the EdU proliferation assay is sensitive and advantageous compared to [3H]-Thymidine radioactive labels in studies on cell proliferation in vitro and allows simultaneous identification of T cell populations. However, this assay has not been established using primary chicken cells to evaluate T cell proliferation by flow cytometry.

**Results:**

Here, we established an assay to evaluate the proliferation of primary chicken splenocytes based on the incorporation of a thymidine analog (EdU) and a click reaction with a fluorescent azide, detected by a flow cytometer. We also established a protocol that combines EdU incorporation and immunostaining to detect CD4^+^ and CD8^+^ proliferating T cells. By inducing cell proliferation with increasing concentrations of a mitogen (Concanavalin A), we observed a linear increase in EdU positive cells, indicating that our protocol does not present any deficiency in the quantity and quality of reagents that were used to perform the click reaction.

**Conclusions:**

In summary, we established a reliable protocol to evaluate the proliferation of CD4^+^ and CD8^+^ chicken T cells by flow cytometry. Moreover, as this is an in-house protocol, the cost per sample using this protocol is low, allowing its implementation in laboratories that process a large number of samples.

## Background

The poultry sector is growing in many countries, with chickens being the largest segment of the industry. However, these birds are exposed to different kinds of stresses and diseases that negatively impact their welfare and consequently, the economy of the industry; therefore many studies have focused on the development of biological products that improve their immune system [1–5]. The chicken immune system functions on the same general principles as the mammalian immune system and is divided into two arms: humoral immunity and cellular immunity [6]. Both CD8^+^ and CD4^+^ T cell subsets, which are components of cellular immunity, are present in chickens [6, 7]. CD8^+^ T cells are the effector cells in cytotoxic responses that kill infected target cells, whereas CD4^+^ T cells collaborate in pathogen elimination [8]. Evidence in the literature indicates that CD4^+^ T cells can also differentiate into Th1 or Th2 cells that are important for the elimination of intracellular or extracellular pathogens, respectively [9]. Many researchers evaluate T cell proliferation to determine whether the cellular immune response is stimulated by a biological product [1, 2, 5, 10]. Therefore, diverse efforts have been realized to standardize techniques allowing this evaluation [11–15]. Proliferation assays are classified into four categories: indirect measures of cell proliferation, cell cycle-associated protein detection, use of cytoplasmic proliferation dyes, and nucleoside-analog [16]. One of the most common methods used with chicken cells is the MTT assay. However, as this assay measures the mitochondrial dehydrogenase activity in cells, and then estimates the cell number from a calibration curve of absorbance versus cell number, its results should be interpreted with caution. Moreover, an increase in cellular mitochondrial activity might not be just an indicator of proliferation as it can be affected by other factors like cell death and decreased or increased metabolic activity of non-proliferating cells [17, 18].

Standardization of flow cytometry-based proliferation assays has been reported and multicolor labeling in these assays allows identification of T cell subsets. The most common flow cytometry-based proliferation assays are dye-based cell proliferation assays and DNA synthesis assays. The CFSE assay, a dye-based cell proliferation assay, has been established with chicken cells [19]. In this assay, a cytoplasmic fluorescent dye, carboxyfluorescein diacetate succinimidyl ester (CFSE), is incorporated into lymphocytes. Subsequently, flow cytometry allows the visualization of each round of division and quantitative analysis of cell division. However, CFSE cell staining, which is performed before cell culture, is cumbersome and during this process, a fraction of cells is lost due to dye toxicity. On the other hand, the most common assay based on DNA synthesis using chicken cells is the 3H-Thymidine (3H-TdR) incorporation assay. However, the main disadvantage of this assay is that the radioisotope (3H-TdR) is a biological hazard to the investigator and environment; further, the assay cannot determine subsets of proliferating lymphocytes. A non-radioactive DNA assay that incorporates the thymidine analog 5′-Bromo-2′-deoxyuridine (BrdU) was established to overcome these limitations. This assay offers an advantage in measuring the cell division of small populations; nonetheless, the major disadvantage of BrdU staining is that the double-stranded DNA blocks the access of the anti-BrdU antibody to BrdU units. Therefore, the samples need to be subjected to denaturing or enzymatic conditions, hindering the detection of the cell surface antigens in chicken lymphocytes [14]. To overcome this, another thymidine analog, ethynyl-deoxyuridine (EdU) has been used. The assay, based on a copper-catalyzed reaction that adds a fluorescent azide to an alkyne group on the DNA-incorporated EdU [20], does not require DNA denaturation for detecting the incorporated nucleoside and it is, thus, possible to identify T cell subpopulations [21, 22]. However, this assay was optimized mainly with mammalian cells and is generally performed using commercial kits. The use of commercial kits increases experimental costs per sample, thereby hindering its implementation in laboratories that process a large number of samples. In this study, we established and optimized an in-house protocol to evaluate the proliferation of spleen mononuclear cells from chickens by flow cytometry. We also included an immunostaining step to identify the T cell proliferation subtype.

## Results

### Optimization of cell culture conditions

**Fig. 1:**
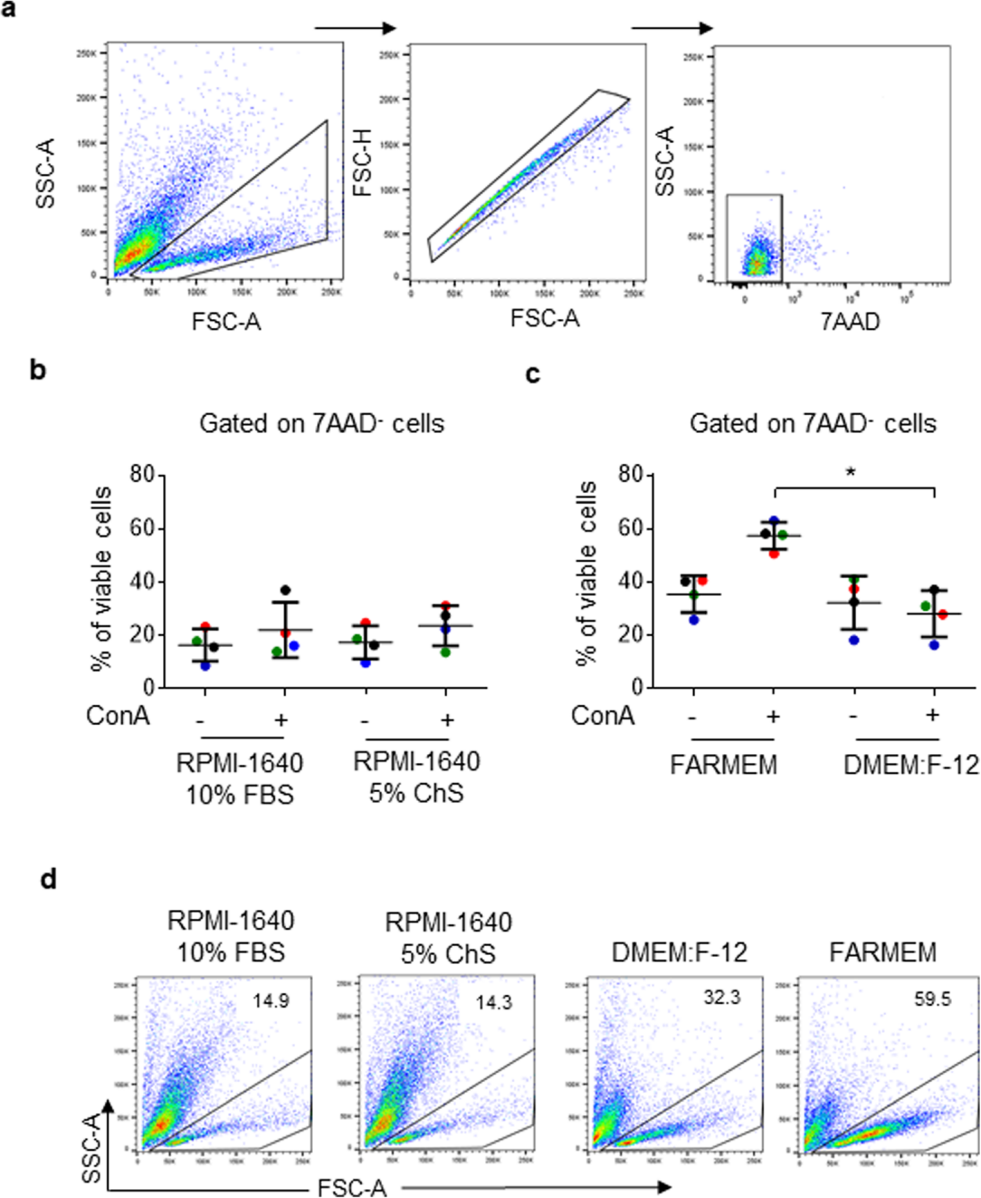
Impact of cell culture medium on cell viability. Spleen mononuclear cells were isolated through a density gradient and cultured in RPMI-1640 supplemented with 5% fetal bovine serum (FBS), RPMI-1640 supplemented with 5% chicken serum (ChS), DMEM:F12 supplemented with 5% ChS, or FARMEM supplemented with 0.5% ChS. Cells were cultured in duplicate in the presence or absence of 1 μg/ml ConA. At 72 h post-plating, cell viability was evaluated using the 7AAD reagent. (**a**) Dot plots representing the flow cytometry analysis. (**b**) Comparison of cell viability in RPMI-1640 supplemented with 5% FBS and RPMI-1640 supplemented with 5% ChS. (**c**) Comparison of cell viability in DMEM:F12 supplemented with 5% ChS and FARMEM. (**d**) Biparametric FSC/SSC dot plot of stimulated cells with ConA in RPMI-1640 supplemented with 5% FBS, RPMI supplemented with 5% ChS, DMEM:F12 or FARMEM medium. Data are represented as the percentage of the 7AAD negative cells in relation to the total population. Results are expressed as the mean ± standard deviation. Significant differences are indicated by * (*p* = 0.0286). Each dot of the same color represents an independent experiment. In each experiment, the cells of 1 chicken were analyzed. Per sample, 30,000 events were acquired on a FACSMelody flow cytometer

Mammalian-suitable media (RPMI-1640 medium) supplemented with chicken serum (ChS) or fetal bovine serum (FBS) are traditionally used for chicken cell culture [11, 12]. However, as shown in Fig. 1b (gating strategy for flow cytometry analysis is depicted in Fig. 1a), we found that spleen mononuclear cells stimulated with the mitogen ConA for 3 days have low viability when cultured in RPMI-1640 medium supplemented with 5% FBS (22 ± 10.8%) or 5% ChS (23 ± 7.47%). As rapidly proliferating cells must acquire more nutrients than non-proliferating cells [23], requiring high levels of glucose and amino acids [24], we evaluated the possibility of improving cell viability using a high-glucose medium like DMEM.F12 (supplemented with 5% of ChS) and a medium that was modified in our laboratory, supplemented with 0.5% ChS (FARMEM medium, industrial secret). As shown in Fig. 1c and d, cell viability was higher using the FARMEM medium than with DMEM.F12 medium (57.7 ± 5.6% vs. 28.4 ± 7.7%; *p* = 0.0286). In this study, we used FARMEM medium due to the high cell viability obtained and the reduction of serum supplementation. Another study recommends the use of X-VIVO™ 15 medium (Lonza, MD, USA), a serum-free medium that reduces the background proliferation of primary chicken cells [19].

### Click reaction and the effect of permeabilization reagents on detection of proliferating cells

**Fig. 2:**
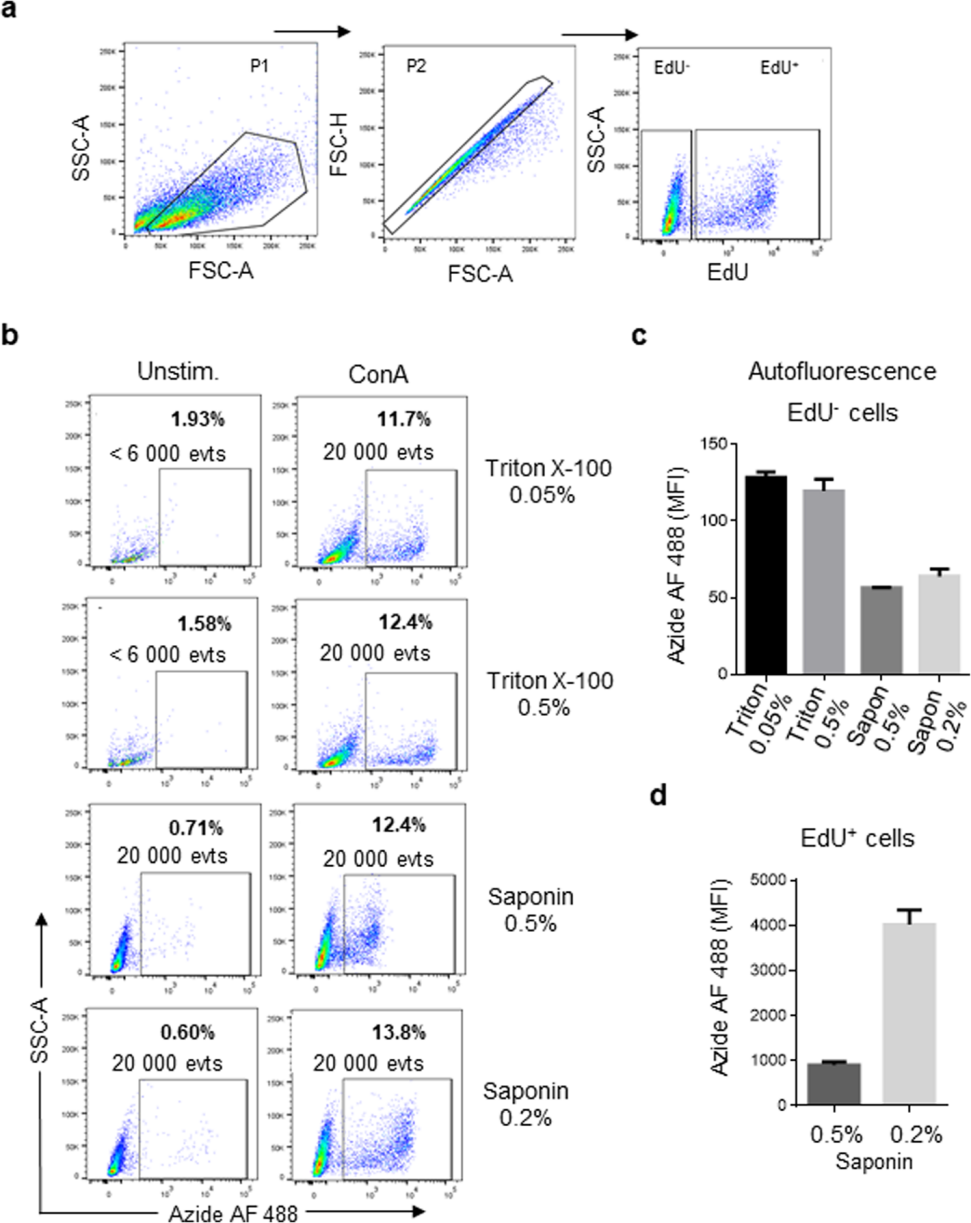
Effect of permeabilization reagents on the detection of EdU^+^ cells. Mononuclear splenocyte cells, cultured for 72 h in the presence or absence of 1 μg/ml ConA, were fixed and treated with different permeabilization reagents (saponin or Triton X-100). (**a**) Flow cytometry analysis for detecting EdU incorporation into cells. (**b**) Representing dot plots of the cells treated with different permeabilization reagents. (**c**) Comparison of median fluorescence intensity (MFI) in EdU^−^ cells (autofluorescence) treated with saponin or Triton X-100. (**d**) Comparison of MFI of EdU^+^ cells treated with 0.5% or 0.2% saponin. Results are the mean ± standard deviation from of 2 independent experiments, performed in duplicate. In each experiment, the cells of one chicken were analyzed. Per sample, 20,000 events were acquired on a FACSMelody flow cytometer

EdU proliferation assay kits from diverse commercial companies are available and validated with mammalian cells. To reduce the cost per sample and validate this protocol with primary chicken cells, we purchased the components of the click reaction from various commercial companies. The click reaction has 3 components: EdU that is incorporated into DNA, fluorescent azide that binds to an alkyne group of EdU through the click reaction, and copper (I) as the catalyst of the click reaction. EdU and Alexa Fluor™ 488 Azide were purchased from Thermo Fisher Scientific (MA, USA) and dissolved in DMSO at 10 mM and 6 mM, respectively. The best EdU staining was reported to be obtained when the click reaction was catalyzed by Cu (I) ions generated from copper (II) sulfate in situ, using ascorbic acid as a reducing agent [20]. So, in this work, we purchased the copper (II) sulfate and ascorbic acid from Sigma-Aldrich Company (MO, USA). As the click components are cell impermeant, we evaluated which permeabilization protocol would allow detection of cells incorporating EdU with low cellular autofluorescence and high recovery of cells post-treatment. Thus, we isolated spleen mononuclear cells and stimulated them with 1 μg/ml ConA for 3 days. Four hours before the end of culture, the cells were incubated with 25 μM of EdU. Subsequently, the cells were fixed with 2% formaldehyde in D-PBS buffer for 10 min at 4 °C, and then the cell membrane was permeabilized with a harsh detergent like Triton X-100 (0.5 and 0.05%) or with a mild detergent reagent like saponin (0.5 and 0.2%), both prepared in D-PBS buffer. The click reaction was carried out under the following conditions described in the literature (the concentration of the fluorescent azide was modified): 6 mM copper sulfate (CuSO_4_), 4 μM Alexa Fluor™ 488 Azide, and 100 mM ascorbic acid [20]. The gating strategy for flow cytometry analysis is shown in Fig. 2a, and as depicted in Fig. 2c and d, the lowest autofluorescence of the EdU^-^ population and the brightest EdU^+^ population was obtained with 0.2% saponin. We also recovered more cells at the end of the process using saponin (Fig. 2b). Thus, we used 0.2% saponin as a permeabilization reagent in subsequent experiments.

### Effects of EdU concentration and EdU incubation time on the click reaction

**Fig. 3:**
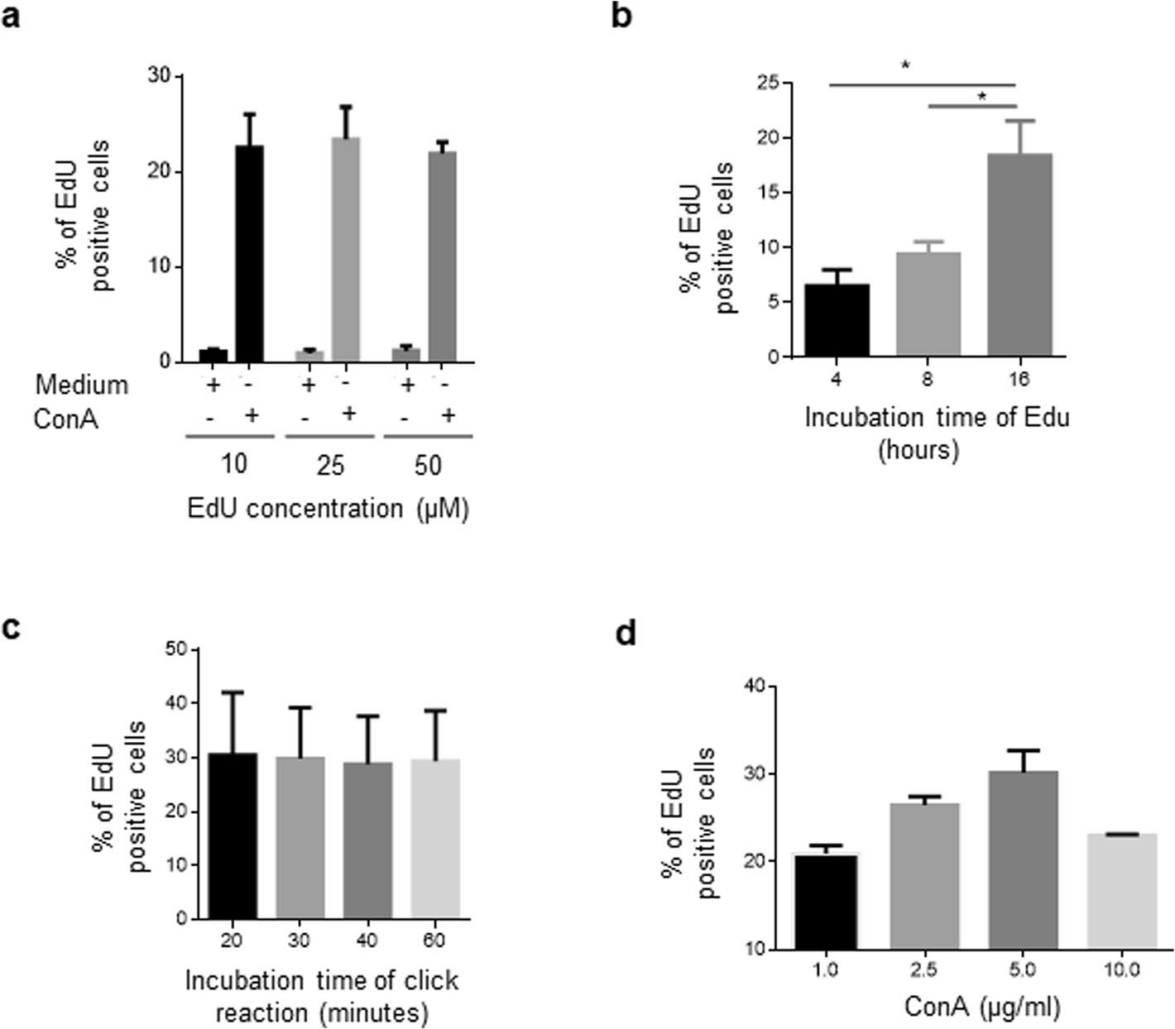
Click reaction conditions. Mononuclear cells isolated from the spleen were cultured in duplicate for 72 h in the presence or absence of 1 μg/ml ConA. The same flow cytometry analysis showed in Fig. 2a was followed. (**a**) Percentage of EdU^+^ cells after 4 h incubation with 10, 25, or 50 μM of EdU. The results are expressed as the mean ± standard deviation of 3 independent experiments. (**b**) Percentage of EdU^+^ cells incubated with 25 μM of EdU for increasing time. The results are expressed as the mean ± standard deviation of 3 independent experiments. (**c**) Percentage of EdU^+^ cells after incubating activated cells with click reaction components for increasing time. The results are expressed as the mean ± standard deviation of 3 independent experiments. (**d**) Percentage of EdU^+^ cells stimulated with increasing concentrations of 1 μg/ml ConA. EdU (25 μM) was added at 16 h before the end of the culture. The staining time with the click reaction solution was 20 min. The results are expressed as the mean ± standard deviation of 2 independent experiments. In each experiment, the cells of 1 chicken were analyzed. All values shown are percentage of singlet cells. Significant differences are indicated by * (*p* = 0.0286). Per sample, 30,000 events were acquired on a FACSMelody flow cytometer

Literature and protocol kits report different EdU concentration and incubation times. Therefore, in the next part of the work, EdU reagent was added for 4, 8, and 16 h prior to cell recovery. As shown in Fig. 3b, at 16 h of EdU incubation, we detected the highest number of proliferating cells compared to that at 8 h (18.4 ± 3.1% vs 9.4 ± 1.2%, *p* = 0.0286) and 4 h (18.4 ± 3.1% vs 6.5 ± 1.5%, p = 0.0286). We also found that EdU reagent can be used in the concentration range of 10–50 μM (Fig. 3a) and that the click reaction is realized in 20 min or less (Fig. 3c). We did not observe an increase in the percentage of proliferating cells in unstimulated conditions (Fig. 3a). To determine whether we were using the right components and concentrations of the click reaction reagents, we stimulated the cells with increasing concentrations of ConA and 16 h before the end of the culture 25 μM EdU was added. As shown in Fig. 3d we observed that the percentage of EdU^+^ cells was increased in a dose-dependent manner, indicating that the click reaction was optimized under the conditions used (25 μM EdU, 4 μM Alexa Fluor™ 488 Azide, 6 mM CuSO_4_, 100 mM ascorbic acid and click reaction time of 20 min).

### Impact of click reaction on the staining of cell surface antigens

**Fig. 4:**
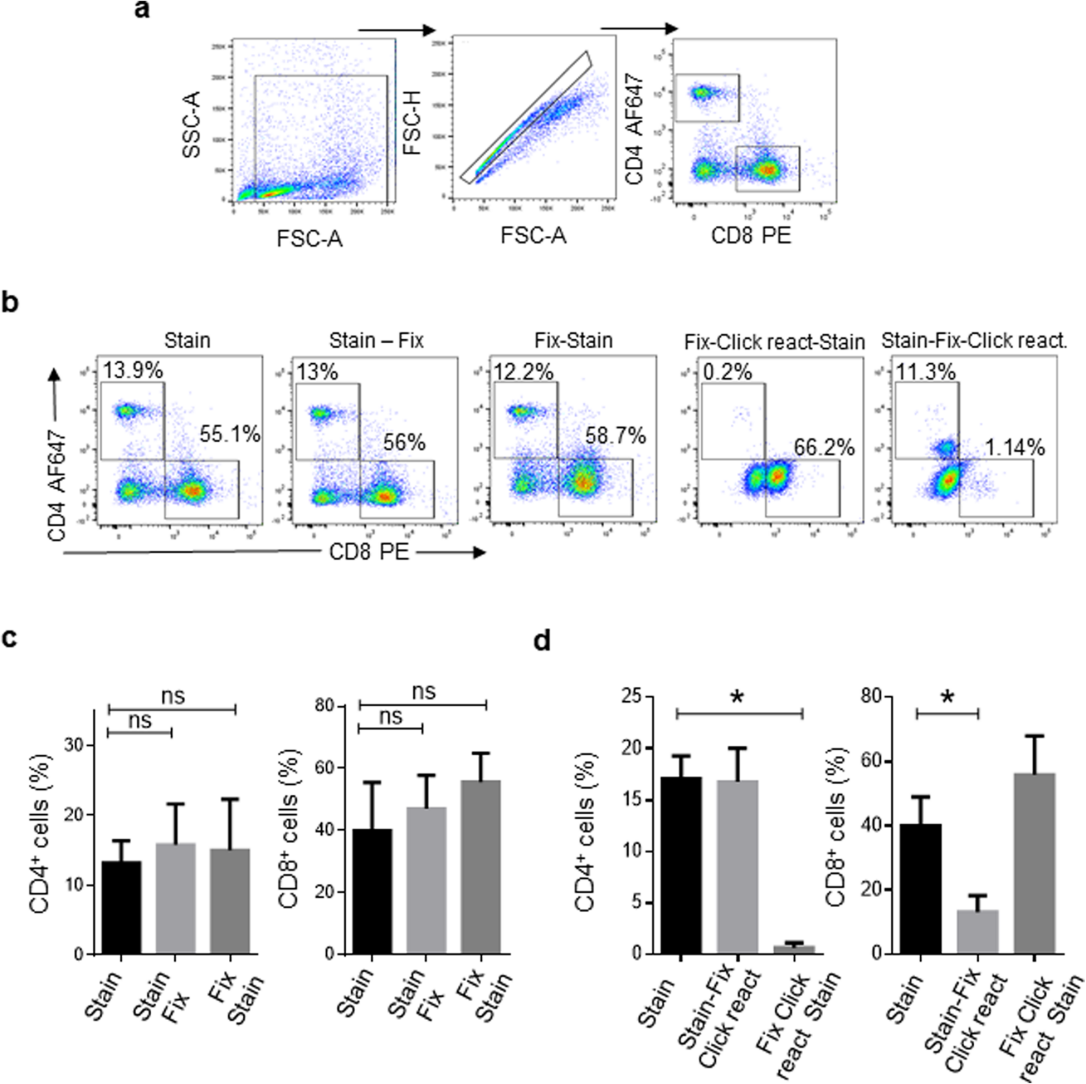
Effect of the fixative and click reaction on antibody fluorescence and T cell surface antigens. Mononuclear cells isolated from spleen of 1 chicken were divided into 5 tubes that received different treatments. (**a**) Dot plots representing flow cytometry analysis. (**b**) Dot plots showing the effect of the fixative and the click reaction on the percentage of CD4^+^ or CD8^+^ cells detected by flow cytometry. (**c**) Quantitative data showing the effect of the fixative on the percentage of CD4^+^ or CD8^+^ cells detected by flow cytometry. The results are expressed as the mean ± standard deviation of 3 independent experiments. (**d**) Quantitative data showing the effect of the click reaction on the percentage of CD4^+^ or CD8^+^ cells detected by flow cytometry. The results are expressed as the mean ± standard deviation of 4 independent experiments. All values shown are percentage of singlet cells. Significant differences are indicated by * (*p* = 0.0286), ns *p* > 0.05. Per sample, 30,000 events were acquired using a FACSMelody flow cytometer

Next, we evaluated the possibility of identifying T cell subpopulations through the use of antibodies. At the beginning of the study, a multicolor panel design was limited by the configuration of the FACSMelody flow cytometer [blue (488 nm) and red (640 nm) lasers]; thus, we used a PE anti-chicken CD8α antibody (clone 3–298) and AF647 anti-chicken CD4 antibody (clone CT-4), clones that were used in other studies [11, 19]. It has been established that prior to the click reaction, a fixation step is necessary to stabilize the cellular membrane. Considering that fixative agents like paraformaldehyde can alter the epitopes or affect the fluorochrome signals, we fixed the cells prior to or after cell staining. As shown in Fig. 4b, c, the percentage of CD4^+^ or CD8^+^ cells was unaltered, indicating that the fixative step does not modify the epitope expression nor the antibody signal (gating strategy for flow cytometry analysis is depicted in Fig. 4a). Subsequently, we evaluated the effect of the click reaction on the surface expression of antigens and the fluorescence signal intensity. As shown in Fig. 4b, d, we barely detect the CD4^+^ population when the click reaction was performed before the staining step, whereas the percentage of the CD8^+^ population was diminished from 40.0 ± 9% to 13.1 ± 5.1% (*p* = 0.0286) when the click reaction was performed after the staining step. As we observed that the click reaction components destroy the CD4 epitope and reduce PE fluorescence, we stained the cells with Alexa Fluor® 647 anti-chicken CD4 antibody before the click reaction and with PE anti-chicken CD8α after the click reaction. Although we detected the CD4^+^ cells and CD8^+^ populations with this dual staining procedure, the PE signal was reduced compared to that in untreated cells. In consequence, it was difficult to separate the CD8^+^ cells from the CD8^−^ cells, resulting in the detection of a higher percentage of CD8^+^ cells after the click reaction than in untreated cells (56 ± 12% vs 40 ± 9%) (Fig. 4d).

**Fig. 5:**
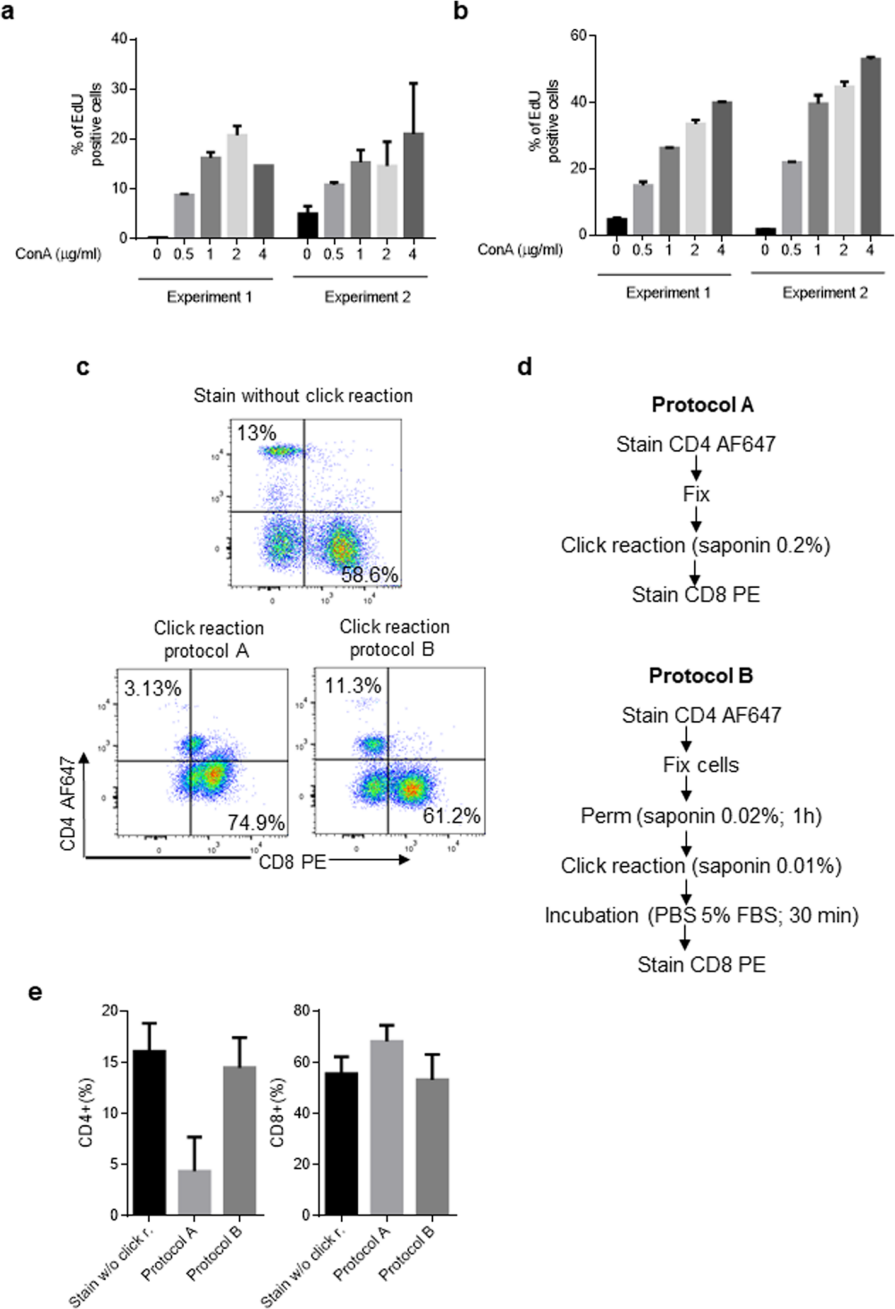
Optimization of the staining protocol. EdU was added 16 h before the end of the culture period. The cells were recovered, fixed, and stained with the click reaction components. (**a**) Spleen mononuclear cells, cultured in the presence of increasing concentrations of ConA (0.5 μg/mL – 4 μg/mL), were subjected to protocol A as described in Fig. 5d. The results are expressed as the mean ± standard deviation of 2 independent experiments. (**b**). Spleen mononuclear cells, culture in the presence of increasing concentrations of ConA (0.5 μg/mL – 4 μg/mL), were treated with protocol B as described in Fig. 5d. The results are expressed as the mean ± standard deviation of 2 independent experiments. (**c**) Dot plots showing the percentage of CD4^+^ and CD8^+^ cells treated with protocol A, protocol B, or from untreated fresh cells. (**d**) Flow chart of the staining process. (**e**) Quantitative data showing the effect of protocol A or B on the percentage of CD4^+^ or CD8^+^ cells as detected by flow cytometry. The results were expressed as the mean ± standard deviation of 3 independent experiments. All values shown are percentage of singlet cells. In each experiment, the cells of one chicken were analyzed. Per sample, 30,000 events were acquired on a FACSCanto II flow cytometer

As CuSO_4_ can affect the PE signal, we reduced the CuSO_4_ concentration from 6 mM to 0.3 mM (the minimal concentration that maintained the correlation showed in Fig. 3d), however, this alteration in the click cocktail did not improve the signal intensity of PE (data not shown). In a study by Xiaojing Sun and collaborators the cell staining was improved upon reducing the saponin concentration [22]. Thus, we reduced the saponin concentration from 0.2% to the concentration that they had used (0.01%). Although this reduction slightly improved the PE signal, we did not observe an increase in the percentage of EdU^+^ cells when they were stimulated with increasing concentrations of ConA (Fig.5a). In order to maintain the positive correlation between the proliferation stimulus and the percentage of EdU^+^ cells, we included a permeabilization step using 0.02% saponin (Fig. 5d). To improve the PE signal, we included an incubation time with 5% FBS (30 min) post the click reaction, as reported by Xiaojing Sun and collaborators [22]. Under these conditions, we observed an increase in the percentage of EdU^+^ cells when stimulated with increasing concentrations of ConA (Fig. 5b), and as depicted in Fig. 5c, e, the PE and AF647 signals were qualitatively improved.

### T cell antigen specific proliferation

**Fig. 6:**
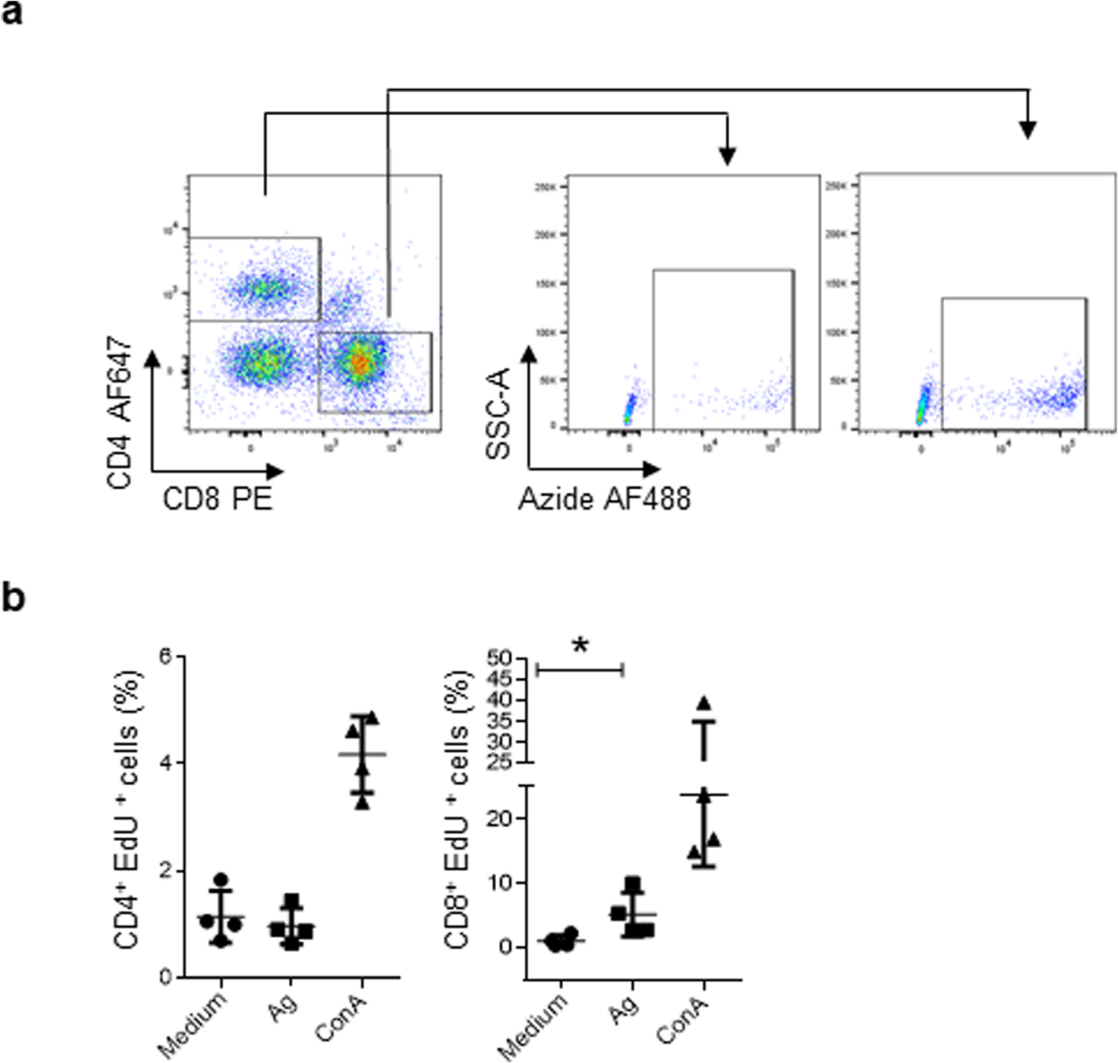
Lymphocyte T proliferation from IBDV immune chickens. Spleen mononuclear cells isolated from 16-weeks old chickens inoculated on day 0 with vectorized vaccine [FARMUNE® (HVT-IBDV-ILTV)], were stimulated in duplicate with 1 μg/ml ConA or 10^7^ copies/ml of inactivated IBDV (infectious bursal disease virus - recall antigen). As basal control, cells were cultured only with medium. (**a**) Flow cytometry analysis protocol used to evaluate the proliferation of CD8^+^ and CD4^+^ cells. (**b**) Quantitative data showing the proliferation of CD4^+^ or CD8^+^ T cells. Each dot represents an animal. All values shown are percentage of CD4^+^ or CD8^+^ cells. The results are expressed as the mean ± standard deviation of one experiment. Significant differences are indicated by *(p = 0.0286). Per sample, 50,000 events were acquired using a FACSCanto II flow cytometer

To demonstrate that this protocol can be used to evaluate antigen-specific proliferation, we cultured spleen mononuclear cells from chickens that were vaccinated against infectious bursal disease virus (FARMUNE®, FARVET, Peru). As observed in Fig. 6, this protocol detected proliferating CD8^+^ T cells that were stimulated in vitro by a recall antigen (inactivated infectious bursal disease virus).

## Discussion

Most studies that evaluate chicken T cell proliferation, as a parameter to determine activation of the cellular immune response, are limited to the MTT or [3H]-Thymidine assays. However, these are old techniques with critical limitations. The application of MTT assay as a technique to evaluate cell proliferation was reported in 1983 [25], whereas the first pitfalls of [3H]-Thymidine incorporation were reported in 1981 [26]. Since then, considerable advances have been made to develop more sensitive and harmless methods that evaluate cell proliferation and identify the cell phenotype. Most of those techniques have been validated using mammalian cells and to our knowledge, only the BrdU and CFSE assays were validated using lymphocyte chicken cells [14, 19]. The BrdU assay is a sensitive technique; however, due to the treatment that the cells are subjected, some surface epitopes can be altered. On the contrary, although the CFSE technique is a sensitive assay that allows identification of the phenotype of proliferating cells, the CFSE dye is toxic to cells even at low concentrations [27]. In our experience, the staining step should be fast to avoid loss of cells due to dye toxicity; thus, another limitation of this technique is that only a small number of samples can be processed simultaneously by a single operator. Other dyes like Cell Trace violet are less toxic; however, in chicken cells, we found poor discrimination between proliferating and non-proliferating cells. In 2007, Adrian Salic and Timothy J. Mitchison developed a method to detect DNA synthesis in proliferating cells [20]. This sensitive method, which is compatible with immunostaining, is based on the incorporation of a thymidine analog (EdU) and its subsequent detection by a fluorescent azide through a Cu(I)-catalyzed cycloaddition reaction (“click” chemistry) [28, 29]. Initially, using this methodology, cellular proliferation was studied through fluorescence microscopy. Thereafter, the assay was adapted to flow cytometry and using commercial kits, it was validated in different samples, including human cells, mice cells, and chick embryos [22, 30, 31]. In this study, we established a protocol to evaluate the proliferation of T cells isolated from chicken spleen. We did not use a commercial kit, as one of the limitations of the technique for its implementation in laboratories is a higher cost per sample. Instead, we purchased the components of the assay from different commercial sources and reduced the cost of consumables per sample. To confirm that our protocol does not present any deficiency in the components of the click reaction, we stimulated the cells with increasing concentrations of ConA. As presented in Figs. 3d and 5b, the percentage of EdU^+^ cells increased in a dose-dependent manner. Like mammals, the main effector cells in chickens are CD3^+^CD4^+^ αβ TCR^+^ T and CD3^+^CD8^+^ αβ TCR^+^ T cells [8]. On the contrary, unlike mice and humans, CD3^+^ γδ TCR^+^ T cells are the major circulating T cell subset and are identified by TCR1 expression [1]. The γδ TCR^+^ T cells proliferate by various stimuli, being their proliferative response dependent on CD4^+^ αβ Tcells [8]. In this study, we established an immunostaining protocol that can be used to detect CD4^+^ and CD8^+^ proliferating T cells. As in the spleen, some CD8^+^ cells also express TCR1 [32], we recommend to include a TCR1 antibody to determine the T cell subtype that is proliferating.

It is also important to mention that in contrast to the CFSE technique that monitors cell proliferation since the beginning of the cell culture, the EdU assay just evaluates it in a short window of time. In consequence, this technique would not be useful to identify slow dividing cells or with a delay in cell proliferation. Another disadvantage is the cytotoxicity of EdU, which makes EdU unusable for long-term experiments [33].

## Conclusion

In summary, we established a reliable protocol to evaluate the proliferation of primary chicken T cells based on the incorporation of EdU (thymidine analog) and their subsequent detection by flow cytometry. The availability of this assay will contribute to advance avian research that needs to evaluate T cell proliferation as a parameter of immune system activation. In addition, as this an in-house protocol, the cost per sample will be lower than that with kits, and its use will be profitable in laboratories that process a large number of samples. The implementation of this technique also will contribute to screening actives components or to the development of new vaccines that improve the chickens’ healthiness, animals that are an important source of protein to humans.

## Methods

### Birds

Twenty-nine specific-pathogen-free (SPF) White Leghorn chickens (layer chickens) of 20-to-40-week-old (Charles River Laboratories, MA, USA) were housed in the SPF area of FARVET company and fed ad libitum with sterilized feed and water. Prior to the experiments, the animals were maintained healthy and were employed to supply the embryonated eggs used in vaccine manufacturing. On the day of the experiment, the animals were euthanized by cervical dislocation without anesthesia following the American Veterinary Medical Association (AVMA) guidelines. The procedure was performed by a trained veterinarian.

Experiments that required statistical analysis were performed 3 or 4 times (mentioned in the figure legends). The cells from 1 animal were analyzed per experiment.

### Isolation and culture of mononuclear spleen cells

Spleens were collected aseptically from the chickens and immediately placed in a tube containing 5 ml of sterile RPMI-1640 medium (Sigma-Aldrich, MO, USA, Catalog # R7755-10 L). Subsequently, the tube was transported on ice to the laboratory. The spleen was perfused with 10 ml of RPMI-1640 supplemented with 10% fetal bovine serum (FBS - HyClone, GE Healthcare, UT, USA, Catalog # SV30180.03). To prepare single-cell suspensions, the splenocytes were strained through a 40 μm mesh into RPMI-1640 culture medium containing 5% FBS. The resulting cell suspension was pelleted by centrifugation for 5 min at 300 ×*g* and resuspended in 4 ml of D-PBS (Sigma Aldrich, Catalog # D5773-50 L). Mononuclear cells were isolated by density gradient centrifugation for 30 min at 400 ×*g* using Histopaque 1.078 (Sigma Aldrich, Catalog # 10771). Then, the cells washed twice with D-PBS (300 ×*g* for 10 min), were resuspended in 2 ml of FARMEM medium (Industrial Secret-FARVET company). An aliquot of cell suspension was mixed with 0.4% trypan blue solution (Sigma-Aldrich, Catalog # 93595-50ML). Through the trypan blue exclusion method, and using a Neubauer chamber the cells were counted, being the cellular viability between 90 and 95%. The cellular concentration was then adjusted to 10 × 10^6^ cells/ml in the FARMEM medium. One hundred microliters of cells were seeded on P96 round-bottom plates and cultured with 5% CO_2_ atmosphere at 41 °C for 3 days in the presence or absence of 100 μl of 1 μg/mL of ConA (Sigma Aldrich, Catalog # C5275). All procedure was performed under sterile conditions in a biosafety cabinet (class II cabinet).

### EdU incorporation

EdU powder was purchased from Thermo Fisher Scientific (MA, USA, Catalog # A10044), dissolved in dimethyl sulfoxide (Sigma Aldrich, Catalog # D4540) at 10 mM concentration, aliquoted, and stored at − 20 °C. EdU previously diluted in cell culture medium was added at a final concentration of 10, 25, or 50 mM at 4, 8, or 16 h before the end of the culture.

### Recovery, fixation, and cell permeabilization

To detach the cells from the plastic, 20 μL of 20 mM EDTA (Calbiochem, CA, USA, Catalog # 324503) in D-PBS buffer (pH 7.4), was added and incubated for 20 min at room temperature [19]. The cells, recovered by pipetting and aspiration, were fixed in 100 μL of 2% formaldehyde (Sigma-Aldrich, Catalog # 1040032500) in D-PBS buffer (pH 7.4) for 15 min at 4 °C and washed twice with 1 ml of D-PBS containing 5% FBS followed by centrifugation of 400 ×*g* for 5 min. To permeabilize the cells with Triton X-100 (Calbiochem, Catalog # 9400), the cells were resuspended in 100 μl of 0.5% or 0.05% Triton X-100 (prepared in D-PBS buffer, pH 7.4) and incubated for 15 min at room temperature. Subsequently, the cells were washed twice with 1 ml of D-PBS and centrifuged at 500 ×*g* for 5 min. Finally, the cells were resuspended in 50 μl of the click staining solution. The saponin reagent (Sigma-Aldrich, Catalog # S7900-100G) was part of the click staining solution, as described in the next section. In the optimized protocol, the fixed cells were permeabilized with 0.02% saponin by 1 h at room temperature. The cells were then washed with 1 ml of 0.02% saponin, centrifuged at 500 ×*g* for 5 min, and resuspended in 50 μl of the click staining solution.

### Click reaction

The components of the Click reaction were as follows: EdU (described above) Copper (II) sulfate (Sigma-Aldrich, Catalog # C3036) diluted in water at 200 mM, Alexa Fluor™ 488 Azide (Thermo Fisher Scientific, Catalog # A10266) reconstituted in dimethyl sulfoxide at 6 mM, and fresh ascorbic acid (Sigma-Aldrich, Catalog # A5960-25G) dissolved in water at 1 M. The optimized staining solution was composed of 0.01% saponin prepared in D-PBS, pH 7.4 (3591 μl of the stock), 0.3 mM copper (II) sulfate (6 μl of the stock), 4 μM Alexa Fluor™ 488 Azide (2.7 μl of the stock), and 100 mM ascorbic acid (400 μl of the stock). The reagents were added in the same order as mentioned above, and the solution was mixed between additions. Subsequently, the fixed cells were resuspended in 50 μL of the staining solution and incubated for 20 min in the dark at room temperature. Finally, the cells were washed twice with 1 ml of D-PBS (500 ×*g*, 5 min). To identify the T cell subset, the cells were resuspended in 300 μl of D-PBS containing 5% FBS and incubated by 30 min in the dark at room temperature. Subsequently, the cells were centrifuged (500 ×*g*, 5 min) and prepared for the staining step.

### Antibodies and flow cytometry reagents

Mouse Anti-Chicken CD4-Alexa Fluor® 647 (clone CT-4, Catalog # 8210–31), Mouse Anti-Chicken CD8α PE (clone 3–298, Catalog # 8405–09) were purchased from SouthernBiotech company (AL, USA). The viability determination reagent, 7AAD, was purchased from BD Biosciences (CA, USA, Catalog # 559925).

### Fluorescent cell staining

Cells were labeled with directly conjugated monoclonal antibodies. Before cell staining, the cells were blocked for 10 min at 4 °C with 15 μl of 2.5% normal mouse serum (Abcam, MA, USA, Catalog # ab7486) prepared in ice-cold D-PBS containing 5% of FBS (FACS buffer). Subsequently, the cells were incubated with 35 μl of Anti-Chicken CD4 or Anti-Chicken CD8α antibodies diluted in ice-cold FACS buffer for 20 min at 4 °C. Cells were then washed with 500 μl of ice-cold FACS buffer followed by centrifugation at 300 ×*g* for 5 min (staining before click reaction) or 500 ×*g* for 5 min (staining after click reaction). Previously for flow cytometric acquisition, the cells were resuspended in 500 μl of FACS buffer and filtered through a 44 μM nylon mesh (Sigma-Aldrich, Catalog # NY4100010) into Falcon® 5 ml polystyrene round-bottom tubes (Corning, NY, USA, Catalog # 352054). All antibodies were titrated to the optimal concentration before use.

### Flow cytometry

Flow cytometry was performed on FACSMelody (BD Biosciences, CA, USA), which is equipped with two lasers: 488 nm and 635 nm, and on FACSCanto II (BD Biosciences, CA, USA), which is equipped with three lasers: 488 nm, 635 nm and 405 nm. Cytometer Performance was checked with CS&T beads (BD) before each acquisition, according to the manufacturer’s instructions.

To establish the EdU protocol 30,000 events were acquired. In antigen-specific stimulation experiments 50,000 events were collected. The data were analyzed using FlowJo software v10.6.1 (BD Biosciences).

### Recall proliferation

Four chickens from 16 weeks of age were inoculated with the vectorized vaccine FARMUNE® (HVT-IBDV-ILTV) on day 0 of age. To prepare the recall antigen, infectious bursal disease virus (IBDV) was obtained from infected cell cultures and was concentrated using a PEG kit (Abcam, Catalog # ab102538) according to the manufacturer’s instructions. Subsequently, the virus was heat-inactivated in a water bath (56 °C, 1 h) aliquoted and stored at − 20 °C. To evaluate the antigen-specific proliferation, spleen mononuclear cells isolated as described above were cultured in presence of 1 × 10^7^ copies/ml of the inactivated IBVD. The IBDV concentration was previously determined by qPCR. To evaluate the basal proliferation the cells were cultured with only medium and as a positive control we used ConA at a final concentration of 1 μg/ml.

### Statistical analysis

All quantitative data were analyzed using the software GraphPad Prism version 6.1 (GraphPad Software, San Diego, CA). Mann-Whitney test was utilized to assess the differences between groups. *p* ≤ 0.05 was considered statistically significant.

## Data Availability

All raw data is stores in the laboratory server and in a cloud service. We will be glad to provide the data, except for the composition of the FARMEM medium (industrial secret). Tables containing the organized data from the study are also available. To be able to access the data please contact Karla Lucia F Alvarez – karla.alvarez@farvet.com or karlalucia220@gmail.com.

## Abbreviations

AF™: Alexa Fluor™
BrdU: Bromodeoxyuridine
CFSE: Carboxyfluorescein succinimidyl ester
ConA: Concanavalin A
ChS: Chicken Serum
DMEM: Dulbecco’s modified eagle medium
DMSO: Dimethyl sulfoxide
DNA: Deoxyribonucleic acid
D-PBS: Dulbecco’s phosphate-buffered saline
EDTA: Ethylenediamine tetra acetic acid
EdU: 5-Ethynyl-2′-deoxyuridine
FACS: Fluorescence activated cell sorting
FBS: Fetal bovine serum
IBDV: Infectious bursal disease virus
HVT: Turkey herpesvirus
ILTV: Infectious laryngotracheitis virus
PBMC: Peripheral blood mononuclear cell
PE: Phycoerythrin
PEG: Polietilenglicol
qPCR: Quantitative polymerase chain reaction
RPMI: Roswell Park Memorial Institute
Th1: T helper 1
Th2: T helper 2
7AAD: 7-Aminoactinomycin D
